# Genomic alterations of cerebrospinal fluid cell-free DNA in leptomeningeal metastases of gastric cancer

**DOI:** 10.1186/s12967-023-04077-8

**Published:** 2023-05-02

**Authors:** Xin Chen, Kaixuan Bai, Yu Zhang, Yang Xu, Yinghao Huo, Sha Wang, Yueli Zou, Xuejiao Qi, Rongyun Guo, Qiuxiang Ou, Dengxiang Liu, Shaohua Yin, Shubo Chen, Hui Bu

**Affiliations:** 1 Department of Neurology, The Second Hospital of Hebei Medical University, 215 Heping West Road, Xinhua District, Shijiazhuang, Hebei Province 050000 People’s Republic of China; 2 Key Laboratory of Neurology of Hebei Province, Shijiazhuang, Hebei 050000 People’s Republic of China; 3 Key Laboratory of Cancer Research, Affiliated Hospital Xingtai People’s Hospital of Hebei Medical University, 818 Xiangdu North Road, Xiangdu District, Xingtai, Hebei 054001 People’s Republic of China; 4 Geneseeq Research Institute, Nanjing Geneseeq Technology Inc., Nanjing, Jiangsu 210032 People’s Republic of China; 5 Department of Neurology, Affiliated Hospital Xingtai People’s Hospital of Hebei Medical University, Xingtai, Hebei Province 054001 People’s Republic of China

**Keywords:** Leptomeningeal metastases, Gastric cancer, Cerebrospinal fluid, ctDNA, Prognosis

## Abstract

**Background:**

Leptomeningeal metastases (LM) were rare in gastric cancer (GC), and GC patients with LM (GCLM) generally suffer from poor prognosis. Nevertheless, the clinical utility of cerebrospinal fluid (CSF) circulating tumor DNA (ctDNA) was underinvestigated in GCLM.

**Methods:**

We retrospectively studied 15 GCLM patients, and all patients had paired primary tumor tissue samples and post-LM CSF samples while 5 patients also had post-LM plasma samples. All samples were analyzed using next-generation sequencing (NGS), and the molecular and clinical features were correlated with clinical outcomes.

**Results:**

CSF had higher mutation allele frequency (*P* = 0.015), more somatic mutations (*P* = 0.032), and more copy-number variations (*P* < 0.001) than tumor or plasma samples. Multiple genetic alterations and aberrant signal pathways were enriched in post-LM CSF, including *CCNE1* amplification and cell cycle-related genes, and *CCNE1* amplification was significantly associated with patients’ overall survival (*P* = 0.0062). More potential LM progression-related markers were detected in CSF samples than in tumor samples, including *PREX2* mutation (*P* = 0.014), *IGF1R* mutation (*P* = 0.034), *AR* mutation (*P* = 0.038), *SMARCB1* deletion (*P* < 0.001), *SMAD4* deletion (*P* = 0.0034), and TGF-beta pathway aberration (*P* = 0.0038). Additionally, improvement in intracranial pressure (*P* < 0.001), improvement in CSF cytology (*P* = 0.0038), and relatively low levels of CSF ctDNA (*P* = 0.0098) were significantly associated with better PFS. Lastly, we reported a GCLM case whose CSF ctDNA dynamic changes were well correlated with his clinical assessment.

**Conclusions:**

CSF ctDNA could more sensitively detect molecular markers and metastasis-related mechanisms than tumor tissues in GCLM patients, and our study sheds light on utilizing CSF ctDNA in prognostic estimation and clinical assessment in GCLM.

**Supplementary Information:**

The online version contains supplementary material available at 10.1186/s12967-023-04077-8.

## Background

The incidence of leptomeningeal metastases (LM) was 5–8% in cancer patients, and patients with LM are usually associated with poor prognosis, suffering from severe symptoms such as headache, nausea, and vomiting. Adenocarcinoma is the major form of LM, and breast, lung, and skin are the most common primary sites of tumorigenesis [1]. In contrast, LM is relatively rare in gastric cancer (GC), with a frequency of only 0.16–0.69% [2–5]. Despite recent advances in systemic chemotherapy, the prognosis of gastric cancer patients with LM remained poor, with a median survival time of 4–6 weeks [6].

Plasma circulating tumor DNA (ctDNA) is tumor-derived cell-free DNA (cfDNA) that was released into the blood circulatory system, and plasma ctDNA has been widely used as a non-invasive tool to characterize tumor genomics [7, 8]. However, in leptomeningeal metastases, plasma ctDNA is in low abundance and present in a limited number of patients. On the other hand, the cerebrospinal fluid (CSF) is in intimate contact with brain malignancies and has been recently demonstrated to contain ctDNA [9]. The CSF space involves the intracerebral ventricles, subarachnoid spaces of the spine and brain (cisterns and sulci), and the central spinal cord canal. Previous studies have reported the detection of tumor genetic alterations using CSF ctDNA in lung cancer patients with LM [9–11]. However, due to limited sample availability, few studies had reported the characteristics of CSF gene mutations in patients with gastric cancer and leptomeningeal metastasis (GCLM).

In the current study, we recruited 15 GCLM patients and conducted next-generation sequencing (NGS) analysis of 425 cancer-relevant genes on matched primary tumor, plasma, and CSF samples. The detected genetic alterations were compared among different sample types, and the molecular results were then correlated with patients’ survival to investigate the prognostic factors related to LM. Overall, our study aimed to elucidate the clinical utility of CSF in GCLM.

## Material and methods

### Patient cohort and samples

A total of 15 GCLM patients were diagnosed and treated in the Department of Neurology, The Second Hospital of Hebei Medical University (Hebei, China) between July 2016 and November 2020. All patients provided signed informed consent and the study protocol was approved by the Research Ethics Committee of The Second Hospital of Hebei Medical University. The LM diagnosis was confirmed using CSF cytology in all 15 patients. Typical imaging of LM was identified as linear or micro-nodular pial enhancement in magnetic resonance imaging (MRI) by two experienced radiologists. Approximately 5 mL of CSF from each patient was collected via lumbar puncture for cytology examination and NGS. Meanwhile, 10 mL blood samples were collected for control and plasma ctDNA detection. Fifteen primary stomach lesion samples were available from the specimen repository. All primary tumor tissue, post-LM plasma samples, and post-LM CSF samples underwent NGS of 425 cancer-relevant genes, and white blood cells were used as normal controls to filter out germline mutations (Additional file 1: Fig. S1). An external cohort of 293 gastric patients from The Cancer Genome Atlas (TCGA) database was used for further data validation, and the detailed clinicopathological features of the validation cohort can be found in Additional file 1: Table S1.

### Preparation of tissue DNA, plasma cfDNA, and CSF cfDNA

Formalin-fixed paraffin-embedded (FFPE) tumor, freshly frozen CSF, and whole blood samples were collected for genomic profiling. All FFPE tissues were reviewed by histopathological assessment. Tissue DNA was extracted using the QIAamp DNA FFPE tissue kit (Qiagen, Germantown, MD, USA) following the manufacturer’s instructions. Similar procedures were used for cfDNA extraction from whole blood and CSF. Total DNA from plasma and freshly frozen CSF was extracted by QIAamp Circulating Nucleic Acid Kit (Qiagen, Germantown, MD, USA). The quantity and quality of the extracted DNA were evaluated using a Qubit 3.0 fluorometer and Nanodrop 2000, respectively (Thermo Fisher Scientific).

### NGS library preparation and sequencing data analysis

Sequencing libraries were prepared using the KAPA Hyper Prep Kit (KAPA Biosystems) according to the manufacturer's suggestions for different sample types. In brief, 1 μg of fragmented genomic DNA underwent end-repairing, A-tailing, and ligation with indexed adapters sequentially, followed by size selection using Agencourt AMPure XP beads (Beckman Coulter). Hybridization-based target enrichment was carried out with the Geneseeq Prime™ panel (Nanjing Geneseeq Technology Inc., Nanjing, JiangSu, China) covering 425 cancer-associated genes, and xGen Lockdown Hybridization and Wash Reagents Kit (Integrated DNA Technologies). Captured libraries by Dynabeads M-270 (Life Technologies) were amplified in KAPA HiFi HotStart ReadyMix (KAPA Biosystems) and quantified by qPCR using the KAPA Library Quantification Kit (KAPA Biosystems) for sequencing. Enriched libraries were sequenced on HiSeq X10 sequencing system (Illumina, San Diego, CA, USA) with 150 bp pair-end reads.

Sequencing data were processed as previously described [12]. In brief, the data was first demultiplexed and subjected to FASTQ file quality control to remove low-quality data or N bases. Qualified reads were mapped to the reference human genome hg19 using Burrows-Wheller Aligner and Genome Analysis Toolkit (GATK 3.4.0) was employed to apply the local realignment around indels and base quality score recalibration. Picard was used to removing PCR duplicates. VarScan2 was employed for the detection of single-nucleotide variations (SNVs) and insertion/deletion mutations. SNVs were filtered out if the mutant allele frequency (MAF) was less than 1% for tumor tissue and 0.3% for plasma and CSF samples. Common SNVs were excluded if they were present in > 1% population in the 1000 Genomes Project or the Exome Aggregation Consortium (ExAC) 65,000 exomes database. The resulting mutation list was further filtered by an in-house list of recurrent artifacts based on a normal pool of whole blood samples. Parallel sequencing of matched white blood cells from each patient was performed to further remove sequencing artifacts, germline variants, and clonal hematopoiesis. The Copy number alterations were analyzed as previously described [13, 14]. The tumor purities were first estimated using ABSOLUTE [15]. Somatic CN alteration events were assigned based on sample-ploidy values calculated in the FACETS algorithm. Structural variants were detected using FACTERA with default parameters [16]. The fusion reads were further manually reviewed and confirmed on Integrative Genomics Viewer (IGV).

### Statistical analysis

Fisher’s exact test was used to compare the frequency of genetic alterations between groups, and it was carried out using the SPSS for Windows software package (ver. 25.0; SPSS Inc., Chicago, IL, USA). Relative ctDNA abundance was defined as Max ctDNA AF*cfDNA concentration (ng/mL). Progression-free survival (PFS) was defined as the time between diagnosis of LM and disease progression or patient death, and overall survival (OS) was defined as the time between the diagnosis of GC and patient death. Prognosis data were analyzed by Kaplan–Meier curve and log-rank test (analyzed using the survival R package). Statistical analyses were performed using the R (v4.2.1). Two-tailed *P* values that were smaller than 0.05 were considered statistically significant.

## Results

### Characteristics of the patients with GCLM

The clinical characteristics of the enrolled 15 GCLM patients were summarized in Table 1 and Additional file 1: Table S2. The majority of the patients were males (93.3%), and the median age was 60, ranging from 30 to 75 years. The most frequent site of primary gastric cancer was the corpus (46.7%), followed by the cardia (33.3%) and antrum (20.0%). Histological examination showed that all 15 patients were adenocarcinoma, and 75% of the patients were diagnosed with diffuse adenocarcinoma. Most of the patients had advanced disease at the initial diagnosis of gastric cancer, including 10 patients (66.7%) at stage IV and 5 patients (33.3%) at stage III. The liver, lung, bone, and lymph nodes were the metastasis sites prior to central nervous system (CNS) metastasis. Eight patients underwent cranial MRI enhancement, 3 of which were negative, 1 had brain parenchyma enhancement, and 4 had leptomeningeal enhancement. A total of 7 (46.7%) patients were treated with chemotherapy combined with other therapies prior to CNS metastasis. After CNS metastasis, 12 (80.0%) patients were treated solely with chemotherapy, 2 (13.3%) patients received chemotherapy combined with other therapies, and 1 (6.7%) patient did not receive any further treatments. The main neurological symptom of CNS was persistent headache (14, 93.3%), which is likely to be caused by elevated intracranial pressure. The median interval time between the initial diagnosis of gastric cancer and LM was 392 days. Given that CSF cytology is the gold standard for LM diagnosis, we found that all 15 patients had malignant cells in their initial lumbar puncture.

**Table 1:** Clinical characteristics of the 15 patients with GCLM

Characteristics	Number of patients	Percentage of patients (%)
Median age, years (range)	60 (30–75)	
Sex		
Female	1	6.7
Male	14	93.3
Disease stage at diagnosis		
III	5	33.3
IV	10	66.7
Site of primary gastric cancer		
Cardia	5	33.3
Corpus	7	46.7
Antrum	3	20.0
Metastasis sites prior to leptomeningeal		
Liver	1	6.7
Lung	2	13.3
Bone	3	20.0
Lymph nodes	8	53.3
Brain	1	6.7
None	4	26.7
Treatment prior to CNS metastasis		
Naive	4	26.7
Chemotherapy	4	26.7
Chemotherapy combined with other therapies	7	46.7
Treatment after CNS metastasis		
Naive	1	6.7
Chemotherapy	12	80.0
Chemotherapy combined with other therapies	2	13.3
Neurological symptoms of CNS		
Headache	14	93.3
Nausea, vomiting	13	86.7
Dizzy	7	46.7
Vision loss	6	40.0
Hearing loss	4	26.7
Muscular weakness	4	26.7
Lalopathy	3	20.0
Ataxia	2	13.3
Osphyalgia	2	13.3
Neck pain	2	13.3

### The difference in genetic profiles between CSF and primary tumor tissue samples

**Fig. 1:**
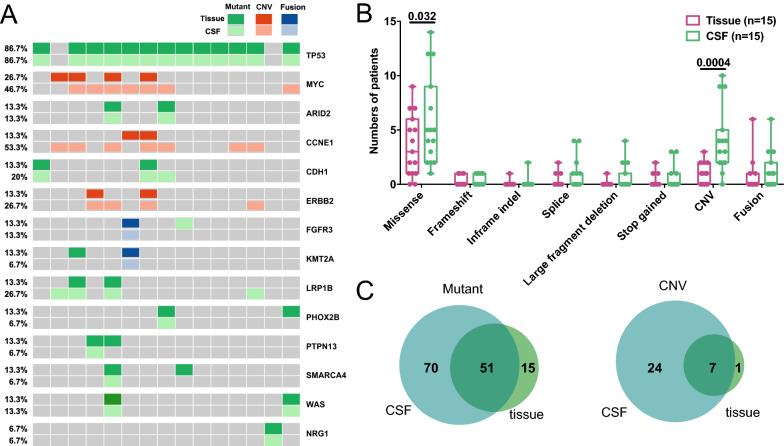
The difference in genetic alterations between CSF and primary tumor tissue samples. **A** The oncoprint plot of paired primary tumor tissue samples and CSF samples in 15 GCLM patients. For each gene, the top row represented primary tumor tissue samples while the bottom row represented CSF samples. **B** The comparison of the type of genetic alterations between primary tumor tissues and CSF. **C** The Venn diagrams of the detected mutations and CNVs between primary tumor tissue and CSF samples

Genetic variations in 15 paired tumor tissue and CSF samples were shown in Fig. 1. *TP53* was the most commonly mutated genes in both tumor tissue and CSF, with 100% detection consistency between the two types of samples (Fig. 1A). The overall median consistency between detected mutations in CSF and tumor tissue in each patient was 83.3% (Additional file 1: Fig. S2A). The amplification of *MYC*, *CCNE1*, and *ERBB2* was more frequently detected in CSF than in tumor tissue samples, with detection rates being 46.7% (7/15) vs 26.7% (4/15), 53.3% (8/15) vs 13.3% (2/15), and 26.7% (4/15) vs 13.3% (2/15), respectively (Fig. 1A). Additionally, CSF harbored significantly more copy number variations (CNVs; *P* < 0.001) and missense mutations (*P* = 0.032) than tumor tissue samples (Fig. 1B), and the mean allele fraction (AF) of all detected mutations was higher in CSF than tumor tissue (*P* = 0.015, Additional file 1: Fig. S2B). A significant proportion of mutations (70/136, 51.5%) and CNVs (24/32, 75.0%) were uniquely detected in CSF samples, while 37.5% (51/136) of mutations and 21.9% (7/32) of CNVs were shared by CSF and tissue (Fig. 1C). In addition, 5 patients (patients 1, 2, 4, 7, and 10) had matched primary tumor tissue, post-LM CSF, and post-LM plasma samples. By analyzing the consistency among the 3 sample types, we found CSF had the highest number of detectable genetic alterations in all 5 patients (Additional file 1: Fig. S2C).

### The potential metastatic mechanism of GCLM

**Fig. 2:**
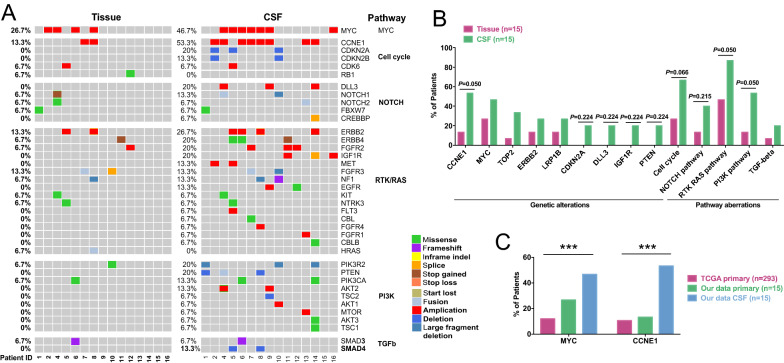
The genetic changes and pathway aberrations enriched after LM. **A** The genetic profile of the pre-LM primary tumor tissue and the matched post-LM CSF samples. **B** The genetic alterations and signaling pathway aberrations that were enriched in post-LM CSF samples when compared with pre-LM primary tumor tissues. **C** The frequency of *MYC* and *CCNE1* amplification among primary tumors in our cohort, primary tumors in the TCGA cohort, and the CSF samples in our cohort

In order to illustrate the potential metastatic mechanism of GCLM, we compared the mutational profile between the paired primary gastric tumor and post-LM CSF samples (Fig. 2A), and we confirmed the findings using an external cohort of primary gastric cancer tumors from the TCGA database. The frequency of *CCNE1* amplification, *MYC* amplification, *CDKN2A* deletion, and *PTEN* deletion was higher in CSF than in primary tumor tissues (Fig. 2A). The pathway analysis revealed that CSF-enriched gene alterations were involved in multiple oncogenic signaling pathways, including the cell cycle pathway (*P* = 0.066), RTK/RAS pathway (*P* = 0.050), and PI3K pathway (*P* = 0.050) (Fig. 2B). The results on *MYC* and *CCNE1* amplification were further recapitulated using the TCGA cohort (Fig. 2C). In particular, among the 293 cases of gastric adenocarcinoma in TCGA database, *MYC* and *CCNE1* amplification was detected in 11.9% and 10.6% patients, respectively, whereas in the CSF samples of our cohort, the *MYC* and *CCNE1* amplification ratios were 46.7% and 53.3%, respectively. Consistently, by using the evolutionary phylogenic analysis, we found *TP53* mutations mainly occurred at the stem region, shared by both post-LM CSF and the primary tumor, whereas *CCNE1* and cell cycle-related genes rapidly evolved in the post-LM CSF branches (Additional file 1: Fig. S3).

### Prognostic factors of GCLM

**Fig. 3:**
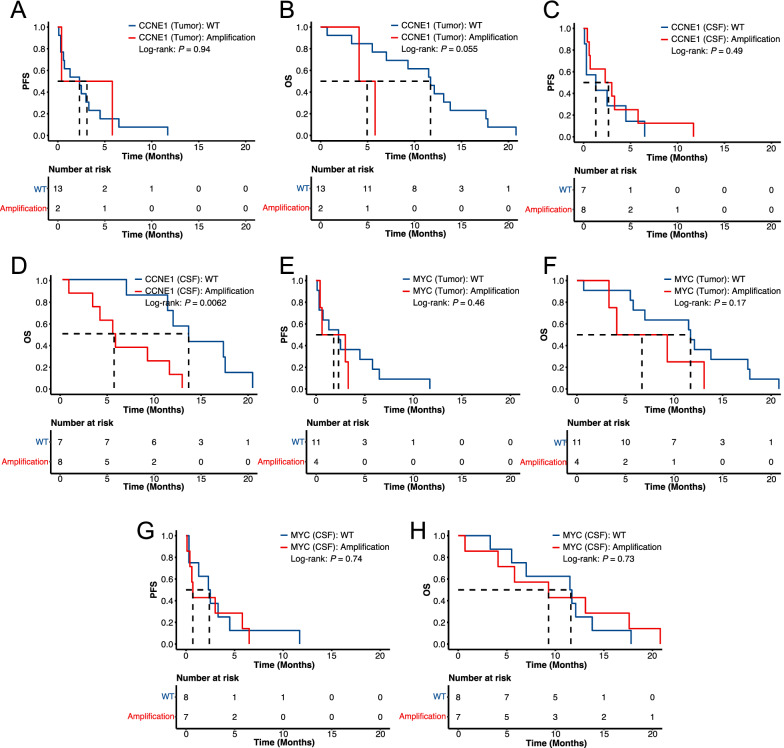
*CCNE1* amplification, but not MYC amplification, was associated with the overall survival of GCLM patients. **A–D** The Kaplan–Meier curves of PFS (**A, C**) or OS (**B, D**) in GCLM patients stratified by *CCNE1* amplification status that was assessed by tumor tissue samples (**A, B**) or CSF (**C, D**). **E–H** The Kaplan–Meier curves of PFS (**E, G**) or OS (**F, H**) in GCLM patients stratified by *MYC* amplification status that was assessed by tumor tissue samples (**E, F**) or CSF (**G, H**). PFS, progression-free survival; OS, overall survival

According to the results of genomic differences between primary tumor and post-LM CSF, we speculated that *MYC* and *CCNE1* amplification may be closely related to LM. We, thereby, performed the Kaplan–Meier and log-rank test analyses using the *MYC* and *CCNE1* amplifications that were assessed by either tumor tissue or CSF samples. Based on the genetic profile of tumor tissue samples, patients with different *CCNE1* amplification statuses had indistinguishable PFS (*P* = 0.94; Fig. 3A), while patients with *CCNE1* amplification tended to have a worse OS (*P* = 0.055; Fig. 3B). Consistently, the CSF-based *CCNE1* amplification had little effects on PFS (*P* = 0.49; Fig. 3C), but it could more significantly separate patients with good and poor OS (*P* = 0.0062; Fig. 3D) when compared with tissue-based *CCNE1* amplification (Fig. 3B). On the other hand, *MYC* amplification seemed to have no statistically significant association with patient’s prognosis (Fig. 3E–H).

**Fig. 4:**
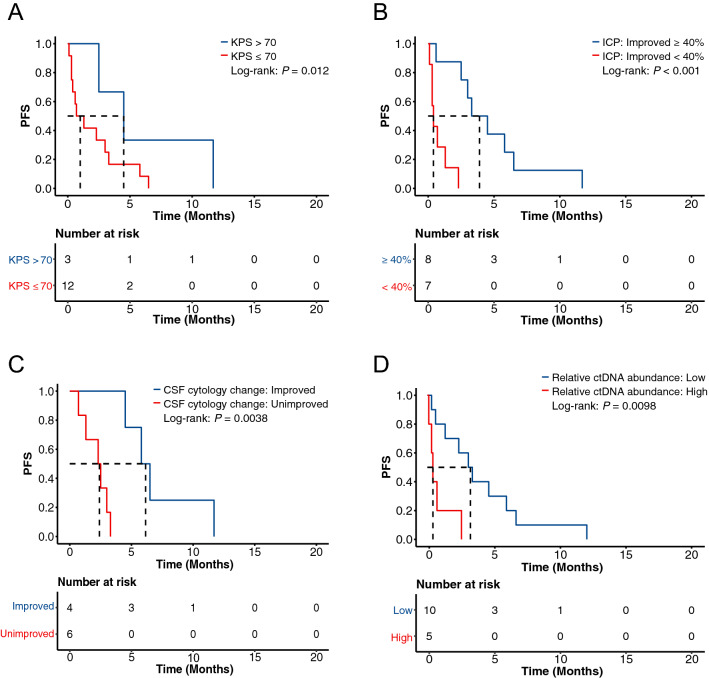
Clinical features that were associated with GCLM disease progression. **A–D** The Kaplan–Meier curves of PFS in GCLM patients stratified by KPS (**A**), ICP improvement levels (**B**), CSF cytology change (**C**), and relative ctDNA abundance (**D**). *PFS* progression-free survival, *KPS* Karnofsky Performance Scale, *ICP* intracranial pressure

Next, we investigated molecular and clinical characteristics that were related to the progression of GCLM. Mutations of *PTPN13* or *ERBB2* in the primary tumor were correlated with significantly shorter PFS (*P* = 0.014 and 0.0034, respectively; Additional file 1: Fig. S4A, B). By contrast, more potential prognostic biomarkers were detected in CSF samples, including *PREX2* mutation (*P* = 0.014), *IGF1R* mutation (*P* = 0.034), *AR* mutation (*P* = 0.038), *SMARCB1* deletion (*P* < 0.001), *SMAD4* deletion (*P* = 0.0034), and TGF-beta pathway aberration (*P* = 0.0038) (Additional file 1: Fig. S4C–H). Additionally, we found that patients with a higher Karnofsky performance scale (KPS) tended to have a longer PFS (HR: 0.45; 95% CI: 0.21–0.97; *P* = 0.12) (Fig. 4A). Also, improvement in intracranial pressure (ICP), improvement in CSF cytology after treatment, and relatively low level of CSF ctDNA were significantly associated with better PFS (Fig. 4B–D). By comparing patients with and without prior LM treatments, we found that there was no significant difference in clinical outcomes and CSF mutational profile (Additional file 1: Fig. S5, Table S3). Overall, multiple molecular and clinical features could be potentially used as prognostic markers for GCLM, and CSF could more sensitively detect these genetic biomarkers than tumor tissue samples.

### Dynamic changes of driver genes in CSF ctDNA throughout therapy for GCLM

**Fig. 5:**
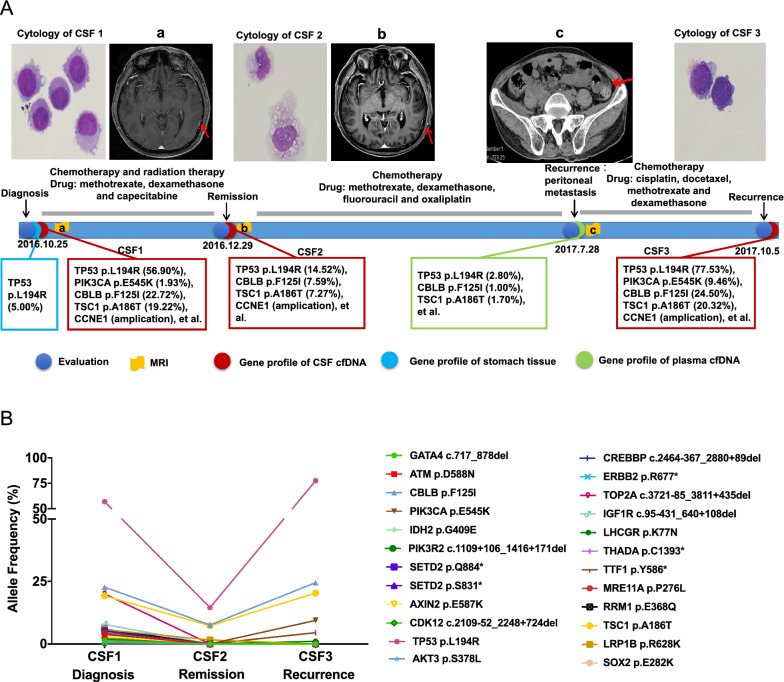
The dynamic change of driver genes in CSF ctDNA throughout therapy in a GCLM case. **A** The treatment history, serial genetic profile from the tumor, plasma, and CSF samples, imaging results, and cytology data of a male GCLM patient. Time point a (CSF 1) represents the time of initial diagnosis on Oct 25, 2016, time point b (CSF 2) represents the time of disease remission on Dec 29, 2016, time point c represents the time of diagnosing peritoneal metastasis on July 28, 2017, and CSF 4 was collected at disease recurrence on Oct 5, 2017. **B** The dynamic change of allele frequency of various mutations detected in serial CSF samples collected during the course of treatment

Lastly, we presented a case to demonstrate the dynamic changes in CSF ctNDA during the course of therapy to treat GCLM. Specifically, a 58-year-old man was admitted to our hospital with a 22-day history of headache, vomiting, dizziness, and left leg weakness. Brain computed tomography (CT) showed no abnormality; however, gadolinium-enhanced T1-weighted MRI of the brain showed leptomeningeal contrast enhancement, which was most prominent over the superior aspect of the temporal lobe (Fig. 5A). CSF cytological analysis revealed the presence of a significant number of malignant cells (Fig. 5A, CSF1), and subsequent gastroscopy detected a poorly differentiated adenocarcinoma at the antrum. Based on this clinical evidence, the patient was diagnosed with leptomeningeal metastasis of gastric cancer and received chemotherapy and radiotherapy. Two months later, his headache symptoms disappeared and CSF cytology showed a decrease in the number of malignant cells (Fig. 5A, CSF2). Despite continued chemotherapy in this patient, peritoneal metastasis was detected 7 months later during a routine review. Severe headaches recurred around 2 months after the diagnosis of peritoneal metastasis. Meanwhile, CSF cytology showed an increase in tumor cells (Fig. 5A, CSF3), so the recurrence of meningeal metastasis was considered. The genetic profile of CSF ctDNA showed dynamic changes in some driver genes. In particular, *CCNE1* amplification was found in CSF samples but not in the corresponding primary tumor samples (Fig. 5A). Besides *CCNE1*, more genetic alterations were detected in CSF samples when compared with primary tumor and plasma samples (Fig. 5A), and the allele frequency dynamics of CSF ctDNA were consistent with the patient's clinical assessment (Fig. 5B), implying the potential clinical utility of CSF ctDNA for GCLM diagnosis and disease monitoring.

## Discussion

GC was a leading cause of cancer-related deaths, especially in developing countries [17]. Liver is the most common site of hematogenous metastasis of GC (4–14%) [18–20], while lung and bone metastases were reported to be around 0.5%-0.96% [21] and 0.9–3.8% [22, 23] in GC patients, respectively. In contrast, LMGC is relatively rare and its incidence rate is only about 0.16%-0.69% [24, 25]. Response to treatment is also extremely poor among LMGC patients, with median OS for GC patients with liver, lung, bone, and leptomeningeal metastasis being 4 months, 3 months, 4 months, and 1.5 months, respectively [6]. In the current study, we used the broad-panel NGS to investigate 15 LMGC patients, and the clinical manifestations and clinical outcomes of our patients were generally consistent with previously reported studies [26, 27]. We found that the mutational profile in CSF ctDNA was generally consistent with those in primary tumors, whereas the average allele frequency of mutations in CSF was significantly higher than that in tumor tissue samples. Additionally, multiple genetic alterations were uniquely identified in CSF ctDNA, particularly some CNV variations that were more frequently detected in CSF, which suggests the importance of CSF ctDNA as a liquid biopsy medium for LM. Indeed, owing to the blood–brain barrier, CSF ctDNA is unable to circulate freely to the blood system, resulting in a limited amount of ctDNA from CNS being released to plasma [28, 29]. Therefore, plasma cannot fully represent the ‘real world’ of intracranial lesions. Lastly, by analyzing patients’ clinical outcomes, we discovered that CSF was more sensitive to detecting prognosis-related biomarkers than tumor tissue samples.

Many previous experimental studies focus on the survival of tumor cells in CSF after leptomeningeal metastasis of solid tumors. Nevertheless, the underlying mechanism of meningeal metastasis of solid tumors is still unclear. Fan et al. reported the potential implication of cell cycle pathway changes in lung cancer leptomeningeal metastasis [30], which is consistent with our results. Cyclin E1, encoded by *CCNE1*, functions together with cyclin-dependent kinase 2 (CDK2) to phosphorylate and inactivate Rb, resulting in activating E2F-mediated transcription and promoting the transition from G1 to S phase to initiate DNA synthesis [31]. *CCNE1* amplification has been reported in breast, ovary, gastric, and endometrial cancers and was related to poor prognosis [32–35]. *CCNE1* amplification is found in 11–12% of gastric cancers, and it was suggested to be associated with liver metastasis in gastric carcinoma [36]. Our data suggest that gene variations in PI3K and cell cycle pathways may promote GCLM, and GCLM patients with *CCNE1* amplification had shorter OS. This indicates that *CCNE1* amplification may serve as a prognostic marker for GCLM, and patients who are positive for *CCNE1* amplification may need to adjust their treatment regimen accordingly.

We discovered that aberrations in SMAD4 or TGF-β pathways were associated with worse clinical outcomes. During cancer progression, TGF-β signaling pathway promotes tumor progression by promoting epithelial-mesenchymal-transition (EMT) invasion and augmenting cellular transformation in advanced stages of malignancy [37]. SMAD4 is a key signaling molecule in TGF-β signaling pathway. In particular, SMAD proteins are phosphorylated and activated by transmembrane serine-threonine receptor kinases in response to TGF-β stimulation. Therefore, we speculate that the activation of EMT and cellular transformation by aberrant TGF-β pathway might underlie the poor prognosis of some GCLM patients, although the results need to be confirmed in future studies.

In our cohort, most patients received intrathecal chemotherapy, some patients received systemic chemotherapy and stereoscopic radiotherapy, while none of the patients received targeted therapy or immunotherapy. The resulting median progression-free survival was 12 weeks. Previous studies found that HER2-positive GC patients had increased incidence of brain metastases [38], and similar results were also observed in HER2-positive breast cancer patients [39]. Several research groups have found that intrathecal trastuzumab was effective against HER2-positive leptomeningeal carcinomatosis for both GC and breast cancer patients [40–42]. In our study, *ERBB2* amplification was detected in the CSF samples of 4 patients, and these patients may thus benefit from HER2-targeted therapies. Additionally, for GC patients without radical surgery or metastatic cancer, comprehensive treatment using systemic anti-tumor drugs has become accepted treatment regimens, including chemical drugs, targeted drugs, and immune checkpoint inhibitors. Notably, anti-PD-1 monoclonal antibody combined with chemotherapy has become the new standard of first-line treatment for advanced metastatic GC. The combination of anti-PD-1 therapy with stereotactic radiosurgery was also shown to be effective in GC patients with brain metastases [43]. In our cohort, 3 patients have high tumor mutation burden, which is a classical biomarker for anti-PD-1/PD-L1 drugs, based on CSF ctDNA analysis, and these patients could potentially be treated with immunotherapies.

There are some limitations of our study. Firstly, the GCLM cohort size was relatively small given the rareness of the samples. Secondly, the study was conducted in a single medical center, so the results of this study need to be validated in future studies. Thirdly, due to the rarity of the sample, we cannot find a good external GCLM patient cohort that contains NGS data of both primary tumor and CSF samples. Fourthly, since both tissue and CSF samples were analyzed by NGS, there might be some potential technical bias in mutation/CNV detection between these two samples. Therefore, future studies using alternative approaches, such as droplet digital PCR (ddPCR), are necessary to validate our data, especially the CSF results. Lastly, our cohort lacked paired post-LM tissue samples and time-series CSF samples, which is important for further validations.

## Conclusions

In conclusion, we performed NGS analyses of matched primary tumor tissue samples, post-LM plasma samples, and post-LM CSF samples from 15 GCLM patients, and we correlated the molecular features with clinical outcomes. We found that CSF could more sensitively detect molecular markers and metastasis-related mechanisms, suggesting a profound potential for using CSF ctDNA in prognostic estimation and clinical assessment in GCLM patients.

## Supplementary Information

**Additional file 1: Fig. S1 **The overview of patient cohorts and the study plan**. Fig. S2** The comparison of different types of cancer samples in 15 GCLM patients. (**A**) The consistency of detected mutations between CSF and tumor tissue samples in each patient. (**B**) The comparison of the mean allele frequency (AF) of all the mutations between CSF and tumor tissue samples. (**C**) The comparison of the genetic mutations among primary tumor, post-LM CSF, and post-LM plasma samples for patients 1, 2, 4, 7, and 10. **Fig. S3** The phylogenetic analysis of the primary tumor and post-LM CSF samples in 15 GCLM patients. For each patient, the shared mutations were shown in black (stem), the primary tumor unique mutations were shown in blue (branch), and the CSF unique mutations were shown in red (branch). **Fig. S4** The molecular characteristics that were associated with GCLM clinical outcomes. (**A-B**) The Kaplan-Meier curves of PFS in GCLM patients stratified by *PTPN13* mutation status (**A**) or *ERBB2* mutation status (**B**) that was assessed by tumor tissue samples. (**C-H**) The Kaplan-Meier curves of PFS in GCLM patients stratified by *PREX2* mutation status (**C**), *IGF1R* mutation status (**D**), *AR* mutation status (**E**), *SMARCB1* deletion status (**F**), *SMAD4* deletion status (**G**), or TGF-beta pathway aberration status (**H**) that was assessed by CSF samples. PFS, progression-free survival. **Fig. S5** The Kaplan-Meier curve of PFS in GCLM patients stratified by the treatment status prior to LM. **Table S1** The clinical characteristics of the 293 primary gastric cancer patients from the TCGA database. **Table S2** The clinical characteristics of the 15 GCLM patients. **Table S3** The comparison of mutation/pathway aberration between patients with and without prior LM treatments.

## Data Availability

The human sequence data generated in this study are not publicly available due to patient privacy requirements but are available upon reasonable request from the corresponding author. Other data generated in this study are available within the article and its Additional files.

## Abbreviations

LM: Leptomeningeal metastases
GC: Gastric cancer
GCLM: GC patients with LM
CSF: Cerebrospinal fluid
ctDNA: Circulating tumor DNA
cfDNA: Cell-free DNA
NGS: Next-generation sequencing
TCGA: The Cancer Genome Atlas
FFPE: Formalin-fixed paraffin-embedded
SNV: Single nucleotide variant
indels: Insertions and deletions
PFS: Progression-free survival
OS: Overall survival
CNS: Central nervous system
KPS: Karnofsky performance scale
HR: Hazard ratio
ICP: Intracranial pressure
CT: Computed tomography
CDK2: Cyclin-dependent kinase 2
EMT: Epithelial–mesenchymal-transition
