# Supplementary material for: Genomic alterations of cerebrospinal fluid cell-free DNA in leptomeningeal metastases of gastric cancer
Source: J Transl Med. 2023 May 2;21:296. doi: 10.1186/s12967-023-04077-8 (PMC10155444; doi:10.1186/s12967-023-04077-8)
Supplement: Supplementary file 1 — Additional file 1: Fig. S1 The overview of patient cohorts and the study plan. Fig. S2 The comparison of different types of cancer samples in 15 GCLM patients. (A) The consistency of detected mutations between CSF and tumor tissue samples in each patient. (B) The comparison of the mean allele frequency (AF) of all the mutations between CSF and tumor tissue samples. (C) The comparison of the genetic mutations among primary tumor, post-LM CSF, and post-LM plasma samples for patients 1, 2, 4, 7, and 10. Fig. S3 The phylogenetic analysis of the primary tumor and post-LM CSF samples in 15 GCLM patients. For each patient, the shared mutations were shown in black (stem), the primary tumor unique mutations were shown in blue (branch), and the CSF unique mutations were shown in red (branch). Fig. S4 The molecular characteristics that were associated with GCLM clinical outcomes. (A-B) The Kaplan-Meier curves of PFS in GCLM patients stratified by PTPN13 mutation status (A) or ERBB2 mutation status (B) that was assessed by tumor tissue samples. (C-H) The Kaplan-Meier curves of PFS in GCLM patients stratified by PREX2 mutation status (C), IGF1R mutation status (D), AR mutation status (E), SMARCB1 deletion status (F), SMAD4 deletion status (G), or TGF-beta pathway aberration status (H) that was assessed by CSF samples. PFS, progression-free survival. Fig. S5 The Kaplan-Meier curve of PFS in GCLM patients stratified by the treatment status prior to LM. Table S1 The clinical characteristics of the 293 primary gastric cancer patients from the TCGA database. Table S2 The clinical characteristics of the 15 GCLM patients. Table S3 The comparison of mutation/pathway aberration between patients with and without prior LM treatments. [file 12967_2023_4077_MOESM1_ESM.docx]

**Supplementary figures**

**
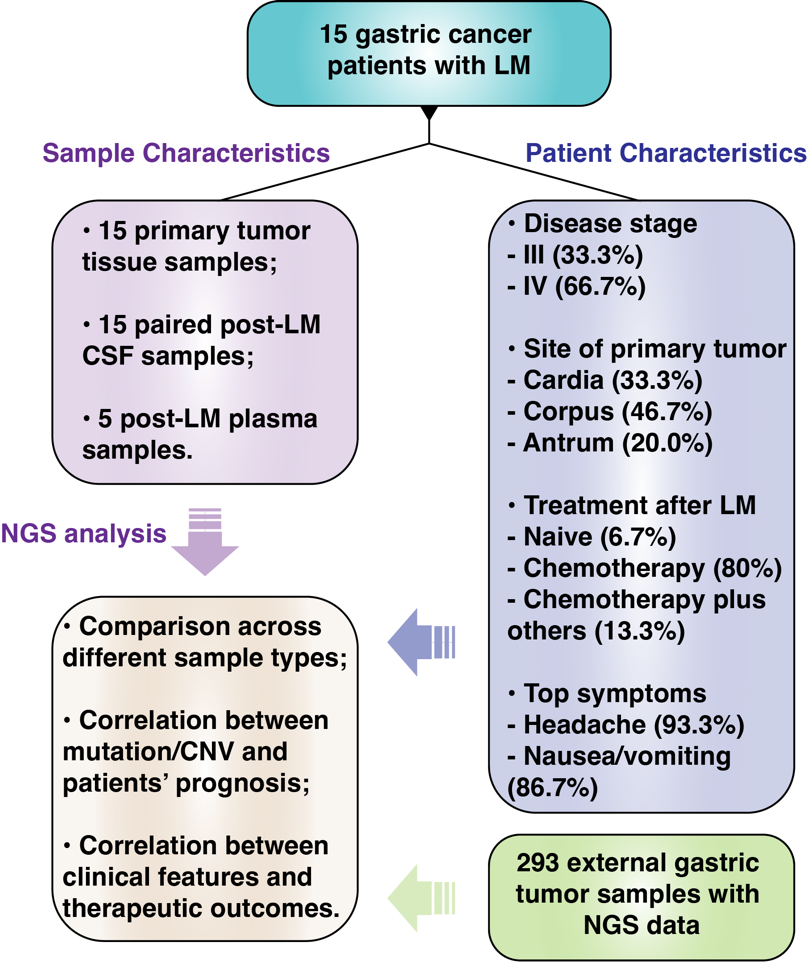
**

**Fig. S1** The overview of patient cohorts and the study plan.

**Fig. S2** The comparison of different types of cancer samples in 15 GCLM patients. (**A**) The consistency of detected mutations between CSF and tumor tissue samples in each patient. (**B**) The comparison of the mean allele frequency (AF) of all the mutations between CSF and tumor tissue samples. (**C**) The comparison of the genetic mutations among primary tumor, post-LM CSF, and post-LM plasma samples for patients 1, 2, 4, 7, and 10.

**Fig. S3** The phylogenetic analysis of the primary tumor and post-LM CSF samples in 15 GCLM patients. For each patient, the shared mutations were shown in black (stem), the primary tumor unique mutations were shown in blue (branch), and the CSF unique mutations were shown in red (branch).

**Fig. S4** The molecular characteristics that were associated with GCLM clinical outcomes. (**A-B**) The Kaplan-Meier curves of PFS in GCLM patients stratified by *PTPN13* mutation status (**A**) or *ERBB2* mutation status (**B**) that was assessed by tumor tissue samples. (**C-H**) The Kaplan-Meier curves of PFS in GCLM patients stratified by *PREX2* mutation status (**C**), *IGF1R* mutation status (**D**), *AR* mutation status (**E**), *SMARCB1* deletion status (**F**), *SMAD4* deletion status (**G**), or TGF-beta pathway aberration status (**H**) that was assessed by CSF samples. PFS, progression-free survival.


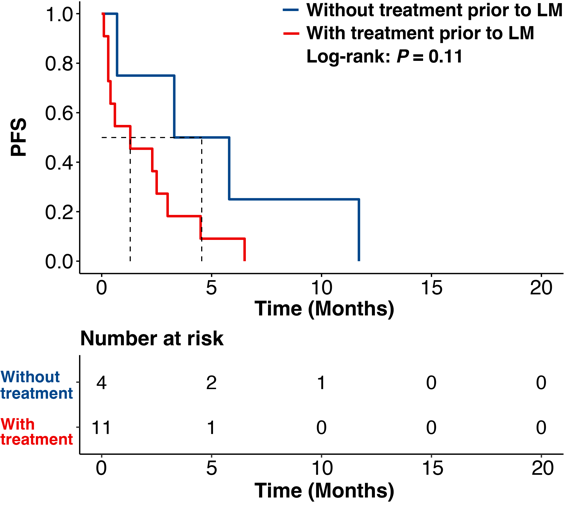


**Fig. S5** The Kaplan-Meier curve of PFS in GCLM patients stratified by the treatment status prior to LM.

**Supplementary tables**

**Table S1** The clinical characteristics of the 293 primary gastric cancer patients from the TCGA database.

| **Characteristics** | **Number of patients** | **Percentage of patients** |
| --- | --- | --- |
| Median age, years (range) | 67.75 (34-90) |  |
| Sex |  |  |
| Female | 113 | 38.6% |
| Male | 180 | 61.4% |
| Histological type |  |  |
| Adenocarcinoma | 293 | 100.0% |
| Stage at diagnosis |  |  |
| I | 32 | 10.9% |
| II | 114 | 38.9% |
| III | 111 | 37.9% |
| IV | 20 | 6.8% |
| Unknow | 16 | 5.5% |

**Table S2** The clinical characteristics of the 15 GCLM patients.

| **Patient ID** | **Sex** | **Age** | **Therapeutic regimens  (primary tumor)** | **Treatment regimens  (after LM)** | **Primary tumor type** | **Tumor site** | **Metastasis prior to LM** | **Disease stage at diagnosis** | **Brain metastasis** | **LM MRI enhancement** | **KPS (pre-treatment)** | **KPS (post-treatment)** |
| --- | --- | --- | --- | --- | --- | --- | --- | --- | --- | --- | --- | --- |
| P1 | Male | 60 | CT+RT | intrathecal CT | adenocarcinoma | corpus | Lymph nodes | 3 | No | NA | 70 | 70 |
| P2 | Male | 60 | naive | intrathecal CT+CT | adenocarcinoma | cardia | Lymph nodes | 3 | No | Negative | 70 | 80 |
| P4 | Male | 66 | CT | intrathecal CT | adenocarcinoma | cardia | Lymph nodes | 4 | No | Positive | 20 | 40 |
| P5 | Male | 60 | CT | intrathecal CT | adenocarcinoma | corpus | Lung | 4 | No | NA | 60 | 30 |
| P6 | Male | 62 | CT+RT | intrathecal CT | adenocarcinoma | cardia | No | 4 | No | Negative | 50 | 50 |
| P7 | Male | 60 | naive | intrathecal CT+CT | adenocarcinoma | cardia | Lymph nodes, bone, and brain | 4 | Yes | Negative | 20 | 40 |
| P8 | Male | 75 | CT | naive | adenocarcinoma and signet-ring cell carcinoma | corpus | Lymph nodes | 4 | No | NA | 50 | NA |
| P9 | Male | 56 | naive | intrathecal CT | adenocarcinoma | corpus | No | 4 | No | NA | 40 | 20 |
| P10 | Male | 69 | Surgery+CT | intrathecal CT | adenocarcinoma | antrum | Lymph nodes | 3 | No | NA | 80 | 90 |
| P11 | Male | 64 | Surgery+CT | intrathecal CT | adenocarcinoma | corpus | No | 3 | No | Negative | 80 | 90 |
| P12 | Male | 30 | CT+immunotherapy | intrathecal CT | adenocarcinoma | corpus | Bone and liver | 4 | No | Positive | 40 | 20 |
| P13 | Male | 62 | CT | intrathecal CT | adenocarcinoma | antrum | No | 3 | No | NA | 60 | 60 |
| P14 | Male | 58 | naive | intrathecal CT+CT+WBRT | adenocarcinoma | antrum | Lymph nodes | 4 | No | Positive | 80 | 90 |
| P15 | Female | 58 | Surgery+CT | intrathecal CT | adenocarcinoma | cardia | Lymph nodes and lung | 4 | No | NA | 50 | 10 |
| P16 | Male | 63 | Surgery+CT | intrathecal CT+Ommaya | adenocarcinoma and signet-ring cell carcinoma | corpus | Bone | 4 | No | Positive | 20 | 40 |

**Table S3** The comparison of mutation/pathway aberration between patients with and without prior LM treatments.

| **Mutations/pathway** | ***P* value** | **Adjusted *P* value** |
| --- | --- | --- |
| AR | 0.26666667 | 1 |
| CSF1R | 0.26666667 | 1 |
| DDR2 | 0.26666667 | 1 |
| ERCC2 | 0.26666667 | 1 |
| FANCE | 0.26666667 | 1 |
| GNAS | 0.26666667 | 1 |
| MCL1 | 0.26666667 | 1 |
| PBRM1 | 0.26666667 | 1 |
| TEK | 0.26666667 | 1 |
| ARID2 | 0.47619048 | 1 |
| CCNE1 | 0.47619048 | 1 |
| FGFR3 | 0.47619048 | 1 |
| KMT2A | 0.47619048 | 1 |
| PHOX2B | 0.47619048 | 1 |
| TP53 | 0.47619048 | 1 |
| RTK.RAS | 0.56923077 | 1 |
| Cell.cycle | 1 | 1 |
| Cell.cycle_activited | 1 | 1 |
| NOTCH | 1 | 1 |
| NOTCH_activited | 1 | 1 |
| PI3K | 1 | 1 |
| PI3K_activited | 1 | 1 |
| TGF_Beta | 1 | 1 |
| TGF_Beta_activited | 1 | 1 |
| RTK.RAS_activited | 1 | 1 |
| WNT | 1 | 1 |
| ABCB1 | 1 | 1 |
| APC | 1 | 1 |
| ARID1A | 1 | 1 |
| BMPR1A | 1 | 1 |
| BUB1B | 1 | 1 |
| CDH1 | 1 | 1 |
| CDK12 | 1 | 1 |
| CDK6 | 1 | 1 |
| CEBPA | 1 | 1 |
| EPHA3 | 1 | 1 |
| ERBB2 | 1 | 1 |
| ERBB4 | 1 | 1 |
| FBXW7 | 1 | 1 |
| FGFR2 | 1 | 1 |
| GRM3 | 1 | 1 |
| HRAS | 1 | 1 |
| KIT | 1 | 1 |
| LRP1B | 1 | 1 |
| MAP3K1 | 1 | 1 |
| MUTYH | 1 | 1 |
| MYC | 1 | 1 |
| NF1 | 1 | 1 |
| NKX2_1 | 1 | 1 |
| NOTCH1 | 1 | 1 |
| NOTCH2 | 1 | 1 |
| NRG1 | 1 | 1 |
| NTRK3 | 1 | 1 |
| PAX5 | 1 | 1 |
| PGR | 1 | 1 |
| PIK3CA | 1 | 1 |
| PIK3R2 | 1 | 1 |
| PTPN13 | 1 | 1 |
| RB1 | 1 | 1 |
| SMAD3 | 1 | 1 |
| SMARCA4 | 1 | 1 |
| TERC | 1 | 1 |
| TERT | 1 | 1 |
| TOP2A | 1 | 1 |
| TSHR | 1 | 1 |
| WAS | 1 | 1 |
